# Possible Implication of the CA2 Hippocampal Circuit in Social Cognition Deficits Observed in the Neuroligin 3 Knock-Out Mouse, a Non-Syndromic Animal Model of Autism

**DOI:** 10.3389/fpsyt.2019.00513

**Published:** 2019-07-19

**Authors:** Brijesh Modi, Domenico Pimpinella, Antonio Pazienti, Paola Zacchi, Enrico Cherubini, Marilena Griguoli

**Affiliations:** ^1^European Brain Research Institute (EBRI), Rome, Italy; ^2^Department of Psychology, Sapienza University of Rome, Italy; ^3^National Center for Radiation Protection and Computational Physics, Italian National Institute of Health, Rome, Italy; ^4^Department of Life Sciences, University of Trieste, Trieste, Italy; ^5^Department of Neuroscience, International School for Advanced Studies (SISSA), Trieste, Italy

**Keywords:** NLG3 KO mice, CA2 hippocampal region, sociability and social memory, excitatory/inhibitory dysfunction, CCK-positive neuron

## Abstract

Autism spectrum disorders (ASDs) comprise a heterogeneous group of neuro-developmental abnormalities with a strong genetic component, characterized by deficits in verbal and non-verbal communication, impaired social interactions, and stereotyped behaviors. In a small percentage of cases, ASDs are associated with alterations of genes involved in synaptic function. Among these, relatively frequent are mutations/deletions of genes encoding for neuroligins (NLGs). NLGs are postsynaptic adhesion molecules that, interacting with their presynaptic partners neurexins, ensure the cross talk between pre- and postsynaptic specializations and synaptic stabilization, a condition needed for maintaining a proper excitatory/inhibitory balance within local neuronal circuits. We have focused on mice lacking NLG3 (NLG3 knock-out mice), animal models of a non-syndromic form of autism, which exhibit deficits in social behavior reminiscent of those found in ASDs. Among different brain areas involved in social cognition, the CA2 region of the hippocampus has recently emerged as a central structure for social memory processing. Here, *in vivo* recordings from anesthetized animals and *ex vivo* recordings from hippocampal slices have been used to assess the dynamics of neuronal signaling in the CA2 hippocampal area. *In vivo* experiments from NLG3-deficient mice revealed a selective impairment of spike-related slow wave activity in the CA2 area and a significant reduction in oscillatory activity in the theta and gamma frequencies range in both CA2 and CA3 regions of the hippocampus. These network effects were associated with an increased neuronal excitability in the CA2 hippocampal area. *Ex vivo* recordings from CA2 principal cells in slices obtained from NLG3 knock-out animals unveiled a strong excitatory/inhibitory imbalance in this region accompanied by a strong reduction of perisomatic inhibition mediated by CCK-containing GABAergic interneurons. These data clearly suggest that the selective alterations in network dynamics and GABAergic signaling observed in the CA2 hippocampal region of NLG3 knock-out mice may account for deficits in social memory reminiscent of those observed in autistic patients.

## Introduction

Autism spectrum disorders (ASDs) comprise a heterogeneous group of neurodevelopmental disorders with a strong genetic component, characterized by deficits in verbal and non-verbal communication, impaired social interactions, restricted interests, and stereotyped behaviors (1). The high incidence of these disorders in recent years (1/100 children with males affected four to six times more than females) can be attributed to the improvement of the diagnostic criteria in general, to the increased attention of the medical community, and to environmental and epigenetic factors acting upon a genetically vulnerable background (2). Most of the genes implicated in ASDs encode proteins relevant for synapse formation, transcriptional regulation, and chromatin remodeling (3). Although mutations or deletions in genes encoding for synaptic proteins are relatively rare (4–8), nevertheless they are important since they point to synapses as possible sites of origin of ASDs, which can be considered synaptopathies (9). One class of non-syndromic forms of ASDs has been found to be associated with mutations or deletions in neuroligin genes (Nlgn1-4) (10–12). Neuroligins (NLGs) are postsynaptic adhesion molecules which bind to their presynaptic partners neurexins to functionally couple the postsynaptic densities with the transmitter release machinery, thus contributing to synapses formation and stabilization (9). To study the mechanisms by which these mutations/deletions contribute to ASD, animal models have been generated that recapitulate important aspects of the human disorder.

Previous studies from NLG3^R451C^ knock-in and NLG3-knock-out mice have revealed the presence of circuit-specific and cell-specific synaptic dysfunctions (13–16). Interestingly, both NLG3^R451C^ knock-in and NLG3-knock-out mice exhibit deficits in social interaction/memory reminiscent of those found in autistic patients (13, 17).

Among brain areas comprising the “social brain” the CA2 region of the hippocampus, characterized by peculiar molecular, morphological, and physiological properties (18, 19), has recently emerged as a central structure for social memory processing (20, 21). This region is responsible not only for encoding social memory, namely, the capacity of an animal to recognize a conspecific (20, 21), but also for social aggression (22). Previous data from mice lacking the Nlgn3 gene have focused on the cerebellum (14) and on the CA1 hippocampal synaptic connectivity (23). However, the CA2 hippocampal circuit, selectively related to social cognition, whose impairment constitutes one of the core symptoms of ASDs, has never been explored.

Here, behavioral, *in vivo* and *ex vivo* electrophysiological recordings from NLG3 knock-out mice have allowed identifying alterations of the CA2 hippocampal circuit probably related to deficits in social memory.

## Methods

### Animals

All experiments were performed in accordance with the Italian Animal Welfare legislation (D.L. 26/2014) that implemented the European Committee Council Directive (2010/63 EEC) and were approved by local veterinary authorities, the EBRI ethical committee, and the Italian Ministry of Health (1084/PR15). All efforts were made to minimize animal suffering and to reduce the number of animals used. NLG3 KO mice were obtained from Prof. P. Scheiffele (Biozentrum, Basel). Experiments were performed on offspring male derived from heterozygous mating after 10 backcrossing with C57BL6/J. The experiments were performed and the results were analyzed blindly before genotyping. Control experiments were performed on wild‐type littermates (controls). Genotyping was carried out on tail biopsy DNA by PCR using a standard protocol. At least five male mice for each genotype were used in a given experiment. Western blot analysis from hippocampal lysates confirmed the lack of NLG3 protein in KO mice (**Figure S1**).

### Western Blot

Hippocampi from controls and NLG3 KO mice were homogenized using a lysis buffer containing 50 mM Tris-HCl, pH 7.4, 150 mM NaCl, 1 mM EDTA, 0.5% CHAPS, 1  mM Na_3_VO_4_, 50 mM NaF, and protease inhibitor mixture (Sigma). Equal amounts of total protein extracts were run on a 10% Sodium Dodecyl Sulfate Polyacrylamide Gel (SDS-PAGE). Western blot analysis was performed with the following primary antibodies: an anti-NLG3 polyclonal antibody (1:1,000, Synaptic System, #129 311) and an anti-actin-HRP (1:5,000, Santa Cruz Biotechnology). Anti-NLG3 Primary antibody was revealed by HRP-conjugated anti-rabbit secondary antibody (Sigma) followed by ECL (Amersham Biosciences).

### Slice Preparation

Transverse hippocampal slices (320 μm thick) were obtained from postnatal (P) day P30–P40 old animals, using a standard protocol (24). Briefly, after being anesthetized with an intraperitoneal injection of a mixture of tiletamine/zolazepam (80 mg/kg) and xilazine (10 mg/kg), mice were decapitated. The brain was quickly removed from the skull, placed in artificial cerebrospinal fluid (ACSF) containing the following (in mM): 75 sucrose, 87 NaCl, 2.5 KCl, 1.25 NaH_2_PO_4_, 7 MgCl_2_, 0.5 CaCl_2_, 25 NaHCO_3_, and 25 glucose. After recovery, an individual slice was transferred to a submerged recording chamber and continuously perfused at 33–34°C with oxygenated ACSF at a rate of 3 ml/min. ACSF saturated with 95% O_2_ and 5% CO_2_ containing the following (in mM): 125 NaCl, 2.5 KCl, 1.25 NaH_2_PO_4_, 1 MgCl_2_, 2 CaCl_2_, 25 NaHCO_3_, and 25 glucose. The osmolarity was 310–320 mOsm.

### Electrophysiological Recordings in Slices

Cells were visualized with a 60× water immersed objective mounted on an upright microscope (Nikon, eclipse FN1) equipped with a CCD camera (Scientifica, UK). Whole-cell patch clamp recordings in voltage-clamp mode were performed with a MultiClamp 700B amplifier (Axon Instruments, Sunnyvale, CA, USA). Patch electrodes were pulled from borosilicate glass capillaries (WPI, Florida, USA); they had a resistance of 3–4 MΩ when filled with an intracellular solution containing the following (in mM): 70 CsMeSO_3_, 70 CsCl, 10 Hepes, 0.2 EGTA, 2 MgCl_2_, 4 MgATP, 0.3 MgGTP, and 5 Na-phosphocreatine; the pH was adjusted to 7.2 with CsOH; the osmolarity was 290–300 mOsm. The holding membrane potential value was not corrected for the liquid junction potential of 10 mV (calculated with the Clampex software; Molecular Devices, Sunnyvale, CA, USA). The stability of the patch was checked by repetitively monitoring the input and series resistance during the experiments. Series resistance (10–20 MΩ) was not compensated. Cells exhibiting 15% changes were excluded from the analysis.

Spontaneous GABA_A_-mediated inhibitory postsynaptic currents (sIPSCs) and AMPA-mediated postsynaptic currents (sEPSCs) were recorded in the CA2 region of the hippocampus from a holding potential of −70 mV in the presence of 6-cyano-7-nitroquinoxaline-2,3-dione (CNQX, 10 μM) and picrotoxin (100 μM), respectively. GABAergic currents (IPSCs) were evoked in CA2 principal cells by stimulation of GABAergic inputs with a glass pipette (filled with ACSF) positioned in stratum pyramidale (at 100–200 µM from the patched cell). Cells were recorded from a holding potential of −50 mV, in the presence of CNQX (10 µM) and DL-2-amino-5-phosphonopentanoic acid (DL-APV, 100 µM), to block α-amino-3-hydroxy-5-methyl-4-isoxazolepropionic acid (ဂAMPA) and N-methyl-D-aspartate (NMDA) receptors, respectively. The intensity of stimulation (delivered at 0.1 Hz) was set at around 60% of the maximum response. Synaptic currents mediated by cholecystokinin-positive interneurons were obtained by subtracting from the total current that sensitive to ω-conotoxin GVIA (ω-CTx-GVIA, 1 µM), known to block N type of voltage-dependent calcium channels, responsible for triggering the release of GABA from CCK+ interneurons (25). Synaptic currents mediated by parvalbumin-positive interneurons were obtained by subtracting from the total current that sensitive to ω-Agatoxin (ω-Agtx-IVA, 300 nM), known to block the P/Q types of voltage-dependent calcium channel responsible for triggering the release of GABA from PV+ interneurons (26). Drugs were applied in the bath, and the ratio of flow rate to bath volume ensured complete exchange within 2–3 min.

### *In Vivo* Electrophysiological Recordings From Anesthetized Animals

Mice (P50–70) were anesthetized with i.p. injection of a mixture of tiletamine/zolazepam (Zoletil; 80 mg/kg) and xilazine (10 mg/kg) to induce anesthesia before surgery and during recordings. A craniotomy for recording sites was drilled between −1.6 and −1.7 mm posterior from bregma, and lateral coordinates were adjusted after extracellular mapping to locate the CA2 and CA3 pyramidal cell layers. Temperature was maintained between 36°C and 37°C using a feedback-controlled heating pad (FHC). Recordings were obtained with a Multiclamp 700B amplifier connected to the Digidata 1550 system. Data were acquired with pClamp 10 (Molecular Devices) and analyzed off-line with Clampfit 10.4 (Molecular Devices).

Extracellular recordings of field potentials were obtained with glass electrodes (Hingelberg, Malsfeld, Germany) prepared with a vertical puller PP-830 (Narishige), and the tip was broken to obtain a resistance of 1–2 MΩ. Electrodes were filled with a standard Ringer’s solution containing the following (in mM): 135 NaCl, 5.4 KCl, 5 HEPES, 1.8 CaCl_2_, and 1 MgCl_2_.

Juxtacellular recordings of spontaneous neuronal firing were obtained using glass electrodes (7–10 MΩ) filled with Ringer solution. Bursts behavior (a burst was defined as a sequence of two or more action potentials occurring at frequency ≥20 Hz) (27) was assessed using the features of spike firing pattern illustrated by plots of interspike interval (ISI) distributions. Bursting behavior showed a distribution of ISI skewed on the left. A clampfit algorithm was used to detect bursts from the total events found setting a threshold. The interval for burst detections ranged from 20 to 50 ms.

### Data Analysis

Data were transferred to a computer hard disk after digitization with an A/D converter (Digidata 1550, Molecular Devices). Data acquisition (digitized at 10 kHz and filtered at 3 kHz) was performed with pClamp 10.4 software (Molecular Devices, Sunnyvale, CA, USA). Input resistance and cells capacitance were measured online with the membrane test feature of the pClamp software. Spontaneous EPSCs and IPSCs were analyzed with pClamp 10.4 (Molecular Devices, Sunnyvale, CA, USA). This program uses a detection algorithm based on a sliding template. The template did not induce any bias in the sampling of events because it was moved along the data trace by one point at a time and was optimally scaled to fit the data at each position.

The rise time of the evoked IPSC was estimated as the time needed for 20–80% increase of the peak current response. The decay time was fitted with first order exponential function in the following form:

y (t)=A*e(−t/τ)

where *τ* and *A* are the time constants and relative fractions of the respective components.

### LFP analysis

#### Unsupervised Decomposition of Local Field Potentials (LFPs)

Raw LFPs data were sampled at 10 kHz and decomposed into their elementary signals or intrinsic mode functions (IMFs) using the “Complete Empirical Ensemble Mode Decomposition with Adaptive Noise “CEEMDAN” algorithm (28). One of the main advantages of this algorithm is that it deals well with non-stationary signals, thus diminishing filtering artifacts (such as harmonics and side band-related distortions) caused by convolution filters for cross-frequency coupling analysis (29–31). We extracted theta (5–14 Hz), low (25–55 Hz), and high gamma (56–120 Hz) signals by combining IMFs with mean instantaneous frequencies [see Ref. (32)].

#### Power Spectral Density (PSD) Analysis

Welch’s PSD was computed using MATLAB *pwelch* function. The Hanning (2048) window was chosen in order to achieve frequency resolution of 5 Hz with overlap of 50%.

#### Average Relative Power of Individual Bands

Average relative amplitude of LFP bands was calculated as *z*-score of individual bands, with standard deviation of raw LFP. This gives us the relative amplitude of each band. Average relative power was obtained by taking the square of relative amplitude and dividing it by the length of the signal.

#### Band Occurrence Frequency

To obtain band occurrence frequency, we used *z*-scored amplitude of individual LFP bands. This was obtained using MATLAB function *z-*score. We then counted the number of cycles (*cycle count*) where amplitude exceeded the *threshold* of 2 standard deviations. Dividing the *cycle count* by total duration gives us the band occurrence frequency for a given LFP band in a given recording file. The choice *threshold = 2 standard deviations* was motivated by the observation that amplitudes in a given LFP band is distributed normally and we wanted to include only those cycles in *cycle count* which takes the amplitude beyond the 95th percentile.

#### Spike Time Extraction

To extract spikes from LFPs we used “wave clus,” a MATLAB tool developed by Chaure et al. (33) that filters raw signals using band-pass filter in the range 300 to 4,500 Hz. Spike detection threshold was set at 5 standard deviations.

#### Power During Spikes

We studied variation in power in LFPs preceding and following 1 s of spike time. This time (1 s) was divided into 10 bins of 100 ms each. We then computed the average relative power as described above for each bin. This allows evaluating how LFP power varies before and after a spike.

#### Spike-Triggered Average LFP Analysis

Analysis of the temporal relationship between multi-unit-activity and ongoing activity was analyzed using custom written scripts in Matlab (The MathWorks, Inc., Natick, Massachusetts, United States). Briefly, spikes were extracted using a threshold on the high-passed version of the recorded membrane potential (cut frequency was set at 500 Hz). The threshold was set at 5 times the standard deviation of this signal. To compute the spike-triggered average, we cut 1 s time segment around the occurrence of each spike using a band-pass-filtered LFP signal (cut frequencies were set at 0.2 and 50 Hz). Experiments where multi-unit-activity were either absent or very sparse (below 1 Hz) were excluded from the analysis.

The power spectrum of **Figure 2C** was computed using running windows of 0.5 s and tapers on the high-pass-filtered membrane signal (cut frequency was set at 3 Hz) using Matlab and the toolbox chronux (http://chronux.org/).

### Drugs

Drugs were applied in the bath by gravity by changing the superfusion solution to one differing only in its content of drug(s). Drugs used were the following: CNQX, DL-APV, ω-CTx-GVIA, and picrotoxin purchased from Tocris (UK) and ω-Agtx-IVA from SIGMA (Italy). Stock solutions were made in distilled water and then aliquoted and frozen at −20°C. Picrotoxin and CNQX were dissolved in DMSO. The final concentration of DMSO in the bathing solution was 0.1%. At this concentration, DMSO alone did not modify the membrane potential, input resistance, or the firing properties of CA2 neurons.

### Behavioral Experiments

#### Three Chamber

In order to evaluate sociability and interest in social novelty, we tested animals by using the three-chamber test, adapted from Moy et al. (34). The social testing area was a homemade rectangular, three-chambered box (each chamber was 20 × 40 × 21 cm in size). Dividing walls were made from clear Plexiglas, with rectangular openings (6 × 8.5 cm) allowing access to each chamber. The light intensity was distributed equally in different parts of the apparatus (6 lux). Between trials, the chambers of the arena were cleaned with 70% ethanol to eliminate any lingering smells.

Mice were habituated to handling 5 min a day for 5 days before the test. The day before the test, mice were placed in the empty chamber and allowed to explore for 30 min. On the test day, a 10 min habituation phase in the empty apparatus took place prior to the test phase. The test phase consisted of three consecutive trials. In the first two trials, sociability was assessed. An unfamiliar C57BL7/6J male mouse (stranger 1) was placed in a wire cup (ø 10.5 cm × 10.5 cm h) in one of the side chambers. In the other side chamber, an identical wire cup was placed to ensure that the test mouse had a choice between a novel object and a novel social context. The position of “stranger 1” was alternated between the first and second trials, to prevent side preference. The test mouse was placed in the middle compartment and allowed to explore the entire social test arena for 10 min. The interaction time with the stranger mouse was recorded by the video-tracking system (ANY-maze, Stoelting Co, IL, US). The score for sociability was calculated as the difference between the investigation time for the compartment containing the novel mouse and that for the compartment with the novel object. After a 1 h inter-session interval a second unfamiliar C57BL7/6J male mouse (stranger 2 or novel) was placed into the previously empty wire cage, while “stranger 1” (familiar) remained inside its cup. The subject mouse was given 10 min to explore all three chambers. “Strangers mice” were 4–5 weeks old (juvenile) to exclude any effect due to mutual aggression. The score for social novelty was calculated as the difference between the investigation time for the compartment containing the novel mouse and that for the compartment with the familiar mouse.

#### Open Field

Mice were also tested in the open field for general locomotor activity, anxiety, and willingness to explore. The experimental apparatus consisted of a black rectangular open field (60 cm × 60 cm × 30 cm; Panlab, US). Mice were allowed to acclimate in their home cage to the procedure room for 30 min before the test. The arena was cleaned with 70% ethanol between trials to eliminate any lingering smells. During the test, each animal was placed in the center of the arena and allowed to freely move for 10 min while being recorded with an overhead camera. Mouse activity was then analyzed by an automated tracking system (ANY-maze, Stoelting Co, IL, US) for the following parameters: total ambulatory distance and velocity. A series of 12 × 12 cm zones were identified and used to evaluate the time spent in the center (inner zone) or peripheral zones (outer zone). The outer zone consisted of 12 blocks close to walls, while the inner zone consisted of 9 blocks in the center. Greater time spent in the outer zones of the maze was indicative of amplified anxiety-related behavior (35).

### Tissue Preparation for Immunohistochemistry

Mice (P60–90) were anesthetized with i.p. injection of a mixture of tiletamine/zolazepam (Zoletil; 80 mg/kg) and xilazine (10 mg/kg) and perfused transcardially with ice-cold oxygenated ACSF (pH 7.4) for 2 min, as previously described (36). Brains were rapidly dissected and fixed overnight in 4% paraformaldehyde (PFA) phosphate buffered saline solution (PBS). To identify the tracks of microelectrodes used to record local field potentials *in vivo*, brains were removed after the completion of electrophysiology experiments and were fixed in 4% PFA overnight. After rinsing in PBS, brains were incubated with 30% (wt/vol) sucrose in PBS at 4°C overnight, frosted with dry ice-cold isopentane, and stored at −80°C. Brains were embedded in the OCT compound (Leica, Germany) and sectioned by cryostat (Leica CM1850 UV, Germany; 60-μm-thick coronal sections).

### Immunohistochemistry

Free-floating sections were rinsed in PBS and incubated overnight at 4°C in primary antibody solution (50 mM Tris, 150 mM NaCl, 0.2% Triton X-100, 2% normal goat and donkey serum, pH 7.4) with antibodies anti-PCP4 (rabbit, 1:200;# SIGMA), anti-CCK8 (mouse, 1:500; #ab37274 Abcam) and anti-PV (guinea pig, 1:1,000; #24428, ImmunoStar). Sections were washed 3 times for 15 min in PBS and incubated in secondary antibody solution (50 mM Tris, 150 mM NaCl, 0.05% Triton X-100, 2% normal goat and donkey serum, pH 7.4) for 1 h at room temperature with secondary antibodies raised in goat and donkey (rabbit AlexaFluor-647, 1:400;#A21245, ThermoFisher, US; mouse AlexaFluor-555, 1:500; #A31570; guinea pig AlexaFluor-488, 1:500; #A11073). Sections were washed 3 times for 15 min in PBS, incubated for nuclear staining DAPI (1:2,000; 3 min), and washed in PBS. Slices were mounted on superfrost slides (Thermo Scientific, US) and cover slipped with Fluoromount Aqueous Mounting Medium (SIGMA).

### Image Acquisition

*z*-Stack images (four optical sections, 0.5 µm step size) were recorded of all specimens using a spinning disk (X-Light V2, Crest Optics) microscope Olympus IX73 equipped with a LED light source (Spectra X light Engine, Lumencore, US) and an Optimos camera (QImaging, Canada). Images were acquired using a 60× or 40× objective with numerical aperture of 1.35 and 1.30, which had a pixel size of 108.3 × 108.3 nm^2^ and 106.2 × 106.2 nm^2^, respectively. The excitation wavelengths used were at 408, 470 and 647 nm. The *z*-stacks were done with a motorized stage (HLD117, Prior Scientific, UK) controlled by MetaMorph software (Molecular Devices). All imaging parameters were kept constant among genotypes. Puncta detection and analysis was performed in the CA2 area identified by PCP4 marker on maximum intensity projections created from *z*-stacks using Image J. A minimum of three mice per group was used, and statistical tests were performed using pooled data points from all mice per group.

### Statistical Analysis

Values are given as the mean ± SEM of n experiments. Significance of differences was assessed by Student’s paired or unpaired *t* test, as indicated. A Mann–Whitney test and Wilcoxon rank sum were used for comparison of two groups. Statistical differences were considered significant at *p* < 0.05. Statistical analysis was performed with GraphPad Prism 8.0 software.

## Results

### NLG3 KO Mice Exhibit Deficits in Social Behavior

**Figure 1:**
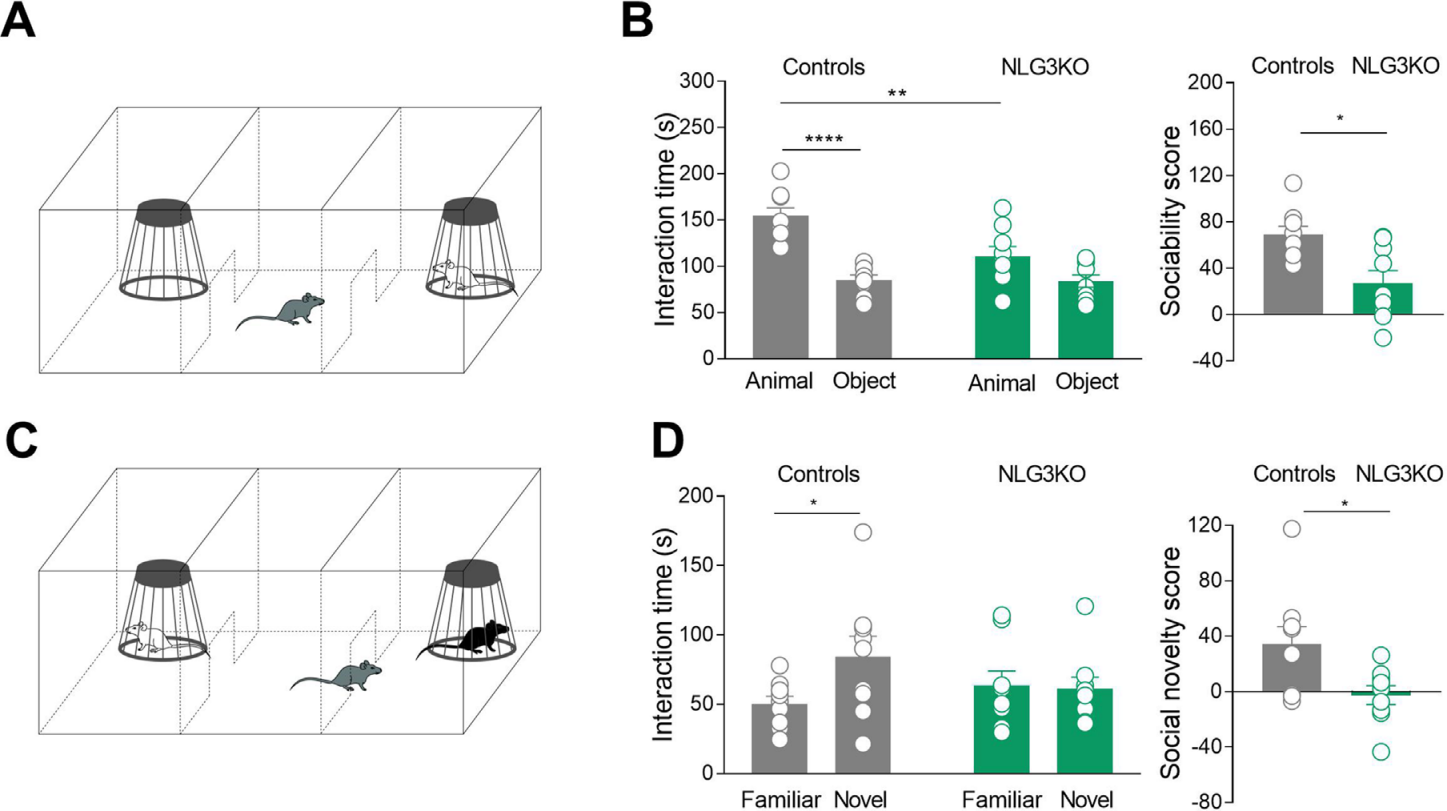
NLG3 KO mice exhibit deficits in social interaction and social memory. **(A)** Schematic representation of three-chamber apparatus to evaluate social interaction. **(B)** Left: scatter plots showing interaction times spent to explore the animal and the object during sociability task in control (gray, *n* = 9) and in NLG3 KO mice (green, *n* = 9); right: scatter plots showing the sociability score in control (gray, *n* = 9) and in NLG3 KO mice (green, *n* = 9). **(C)** Schematic representation of three-chamber apparatus to evaluate social memory (performed 1 h after the sociability test). **(D)** Left: scatter plot showing interaction times spent to explore the novel and the familiar animal in the social novelty task in control (gray, *n* = 9) and in NLG3 KO mice (green, *n* = 9). Right: scatter plot showing the social novelty score in control (gray) and in NLG3 KO mice (green). Open circles are values from single animals, and bars are mean ± SEM.*p < 0.02; **p < 0.001; ****p < 0.0001; Mann–Whitney test.

One of the core symptoms of autism is the impairment in social cognition. Previous studies from NLG3 KO mice have revealed altered social memory, probably related to olfactory deficiency with no alterations in time spent in social interaction (17). However, when tested in the three-chamber apparatus, while control mice exhibited normal levels of sociability, spending more time in exploring the mouse cage with respect to the empty one (object), NLG3 KO mice did not show any preference for the mouse cage (**Figures 1A, B**). The exploration time of mouse cage was significantly lower in NLG3 KO mice as compared to controls (111 ± 10.2 s *vs* 84 ± 6.6 s, *p* = 0.08 Mann–Whitney test and 155 ± 8.4 s *vs* 85.4 ± 5.3 s, *p* < 0.0001, Mann–Whitney test, in NLG3 KO and control mice, respectively) suggesting a reduced interest in investigating a conspecific.

When tested for social novelty, an hour after the sociability test, while control mice spent more time exploring the “novel” than the “familiar” mouse, NLG3 KO mice showed, as in Radyushkin et al., (17), no preference for the novel animal (**Figures 1C, D**). The interaction time was 84.2 ± 15 s *vs* 50.2 ± 5.6 s (*p* = 0.02, Mann–Whitney test) and 61.2 ± 8.4 s *vs* 63.7 ± 10 s (*p* = 0.1, Mann–Whitney test) in control and KO mice, respectively. To understand if the social impairment is associated to locomotor alterations or changes in anxiety levels, we tested both NLG3 KO mice and control littermates in the open field test. According to previous work (37, 38) NLG3 KO mice showed a strong tendency for an increased total ambulatory distance (**Figures S2A, B**; controls: 39.4 ± 3.2 m, *n* = 10; NLG3 KO: 51.7 ± 5.2 m, *n* = 9; *p* = 0.057, unpaired *t* test) and velocity (**Figure S2C;** controls: 65.8 ± 5.3 mm/s; NLG3 KO: 86.2 ± 8.7 mm/s, *n* = 9; unpaired *t* test, *p* = 0.057) as compared to controls. No differences in the time spent in the center (**Figure S2D**; controls: 58.7 ± 5.8 s; NLG3 KO: 61.8 ± 7.8 s; *p* = 0.75, unpaired *t* test) or peripheral (**Figure S2D**; controls: 541 ± 5.8 s; NLG3 KO: 538 ± 7.8 s; *p* = 0.75, unpaired *t* test) zones were detected in both genotypes indicating that there were no changes in anxiety levels. Taken together, these data show a critical role for NLG3 in social behavior including sociability and social memory, reminiscent of those observed in autistic children.

### Impairment of Spike-Related Slow Wave Activity in the CA2 Region of the Hippocampus of NLG3 KO Mice

**Figure 2:**
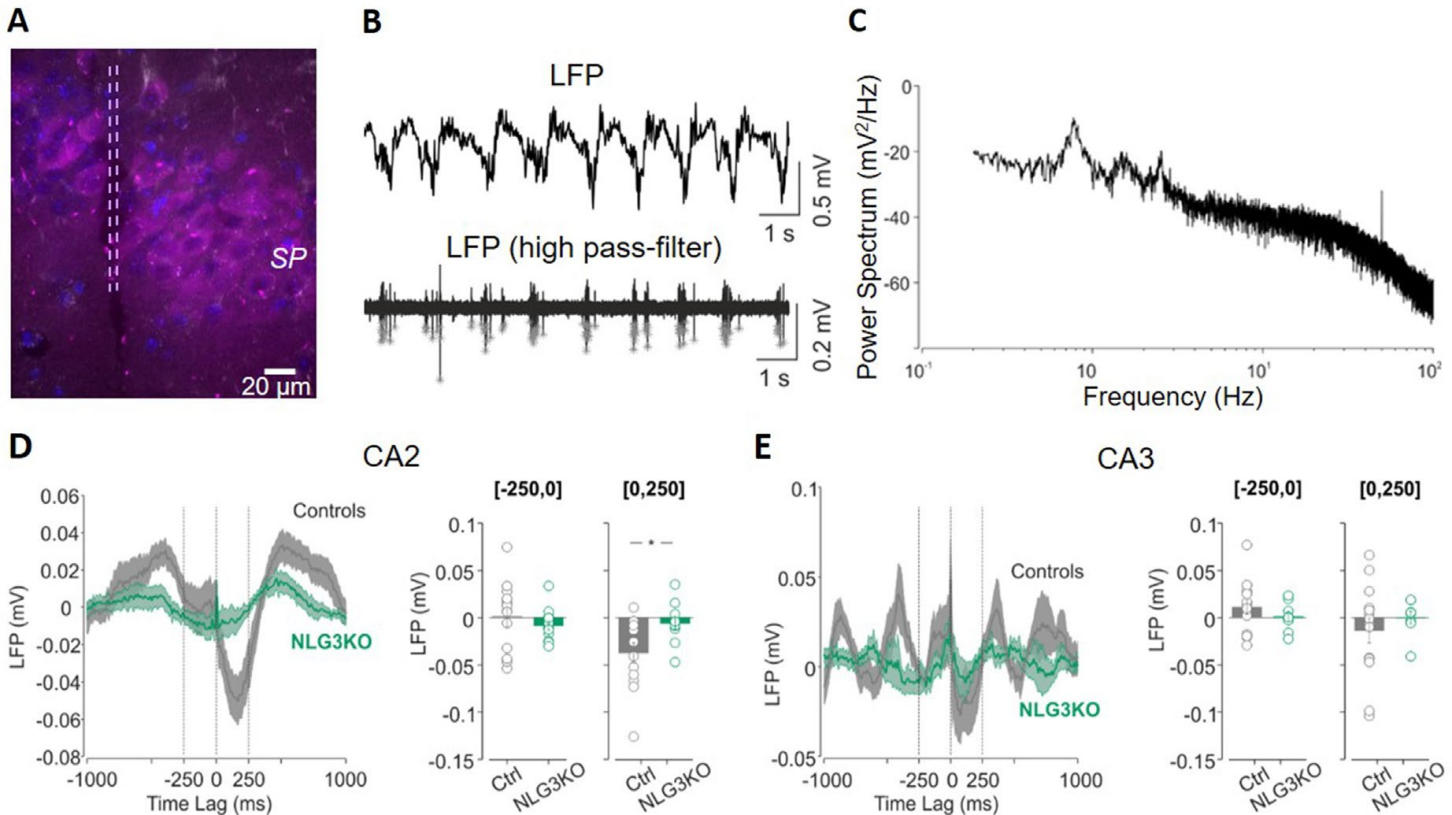
Decreased spike-triggered local field potential (LFP) in the CA2 hippocampal region of NLG3 KO mice. **(A)** Confocal image showing the electrode track (lesion highlighted by dashed lines) in the CA2 pyramidal layer (SP) marked with anti-PCP4 antibody (magenta). **(B)** Samples traces of LFPs recorded in the CA2 region from an anesthetized animal, filtered at 0.2–50 Hz (top) and at 500 Hz (bottom). Gray stars show single action potentials obtained after high-pass filtering. **(C)** Representative power spectrum showing strong oscillations around 1 Hz, typical of anesthesia conditions. **(D)** Left: spike-triggered average of LFPs recorded in area CA2 from control (gray, *n* = 12) and NLG3 KO mice (green, *n* = 12). Average LFP value in the interval preceding (250–0 s, middle) and following (0–250 s, right) the occurrence of the spike. **(E)** Same as **(D)**, but for the CA3 region (control, *n* = 14; NLG3KO, *n* = 8). Bars are mean ± SEM.*p < 0.05, two-sided Wilcoxon rank sum test.

In order to understand whether defects in social behavior observed in NLG3 KO mice depend on alterations in network dynamics, local field potentials (LFPs) were recorded in *in vivo* anesthetized animals from the CA2 (**Figure 2A**) and CA3 hippocampal regions. Firstly, we analyzed the relation between LFPs and multi-unit activity (MUA) recorded in stratum pyramidale. In the presence of anesthetics, LFPs were characterized by slow waves, occurring at the frequency of about 1 Hz (**Figures 2B, C**). By filtering the LFP signals for frequencies above 500 Hz, we could clearly see the signatures of MUA (**Figure 2B**, bottom). We then computed the spike-triggered average (STA) LFP by averaging the LFP waveform in the time window (−1, +1 s) around each MUA (see Methods, **Figures 2D, E**). When calculating the average value of the LFP in the first 250 ms following a MUA, we observed a strong reduction for NLG3KO as compared to control mice for data recorded in the CA2 region (**Figure 2D**, right; −0.034 ± 0.001 mV and −0.006 ± 0.006 mV in control and NLG3 KO, respectively; *p* = 0.02, two-sided Wilcoxon rank sum test). In CA3 recordings, we observed a tendency to weaker oscillations (although not significant) around the MUA, (**Figure 2E**; −0.014 ± 0.013 mV and 0.02 ± 0.007 mV in control and NLG3 KO mice, respectively; *p* = 0.4, two-sided Wilcoxon rank sum test). These results clearly demonstrate that the lack of NLG3 selectively alters synchronization between action potentials and LFP in the CA2 area of the hippocampus.

### Reduced Power of Theta and Gamma Oscillations in Hippocampal Subfields of NLG3 KO Mice

**Figure 3:**
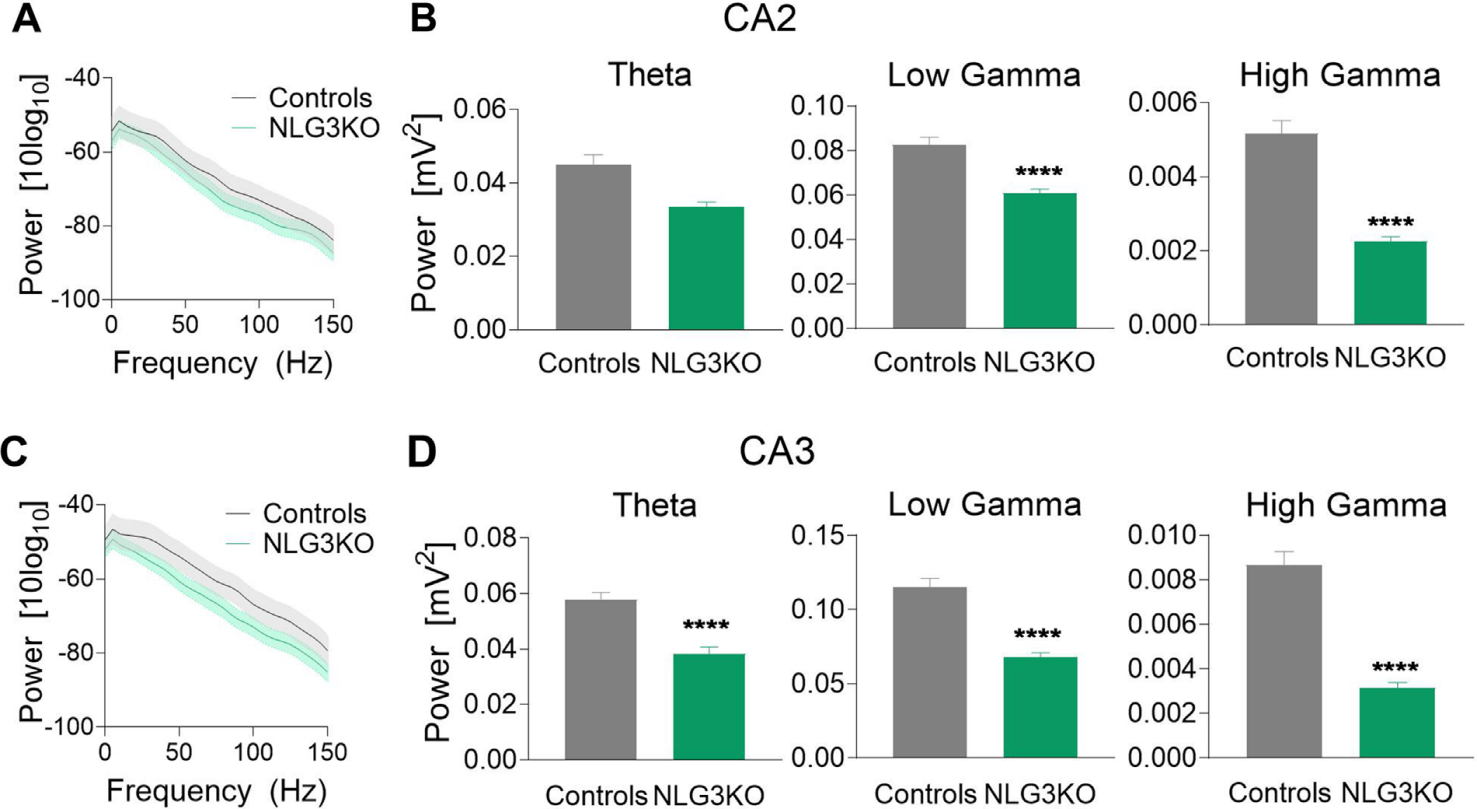
Reduced power in the theta and gamma frequency bands in NLG3 KO mice. **(A)** Welch’s Power spectrum (1–150 Hz) of LFP recorded from the CA2 region of anesthetized control (gray) and NLG3KO (green) mice (shaded areas: SEM). **(B)** Summary plots of relative LFP power in the theta (5–14 Hz, left), the low gamma (25–55 Hz, middle), and high gamma (56–120 Hz, right) bands in controls (gray, *n* = 17) and in NLG3 KO mice (green, *n* = 14). **(C, D)** Same as A, B but for the CA3 area (control, *n* = 15; NLG3KO, *n* = 11). Bars are mean ± SEM.****p < 0.0001, Mann–Whitney test.

**Figure 4:**
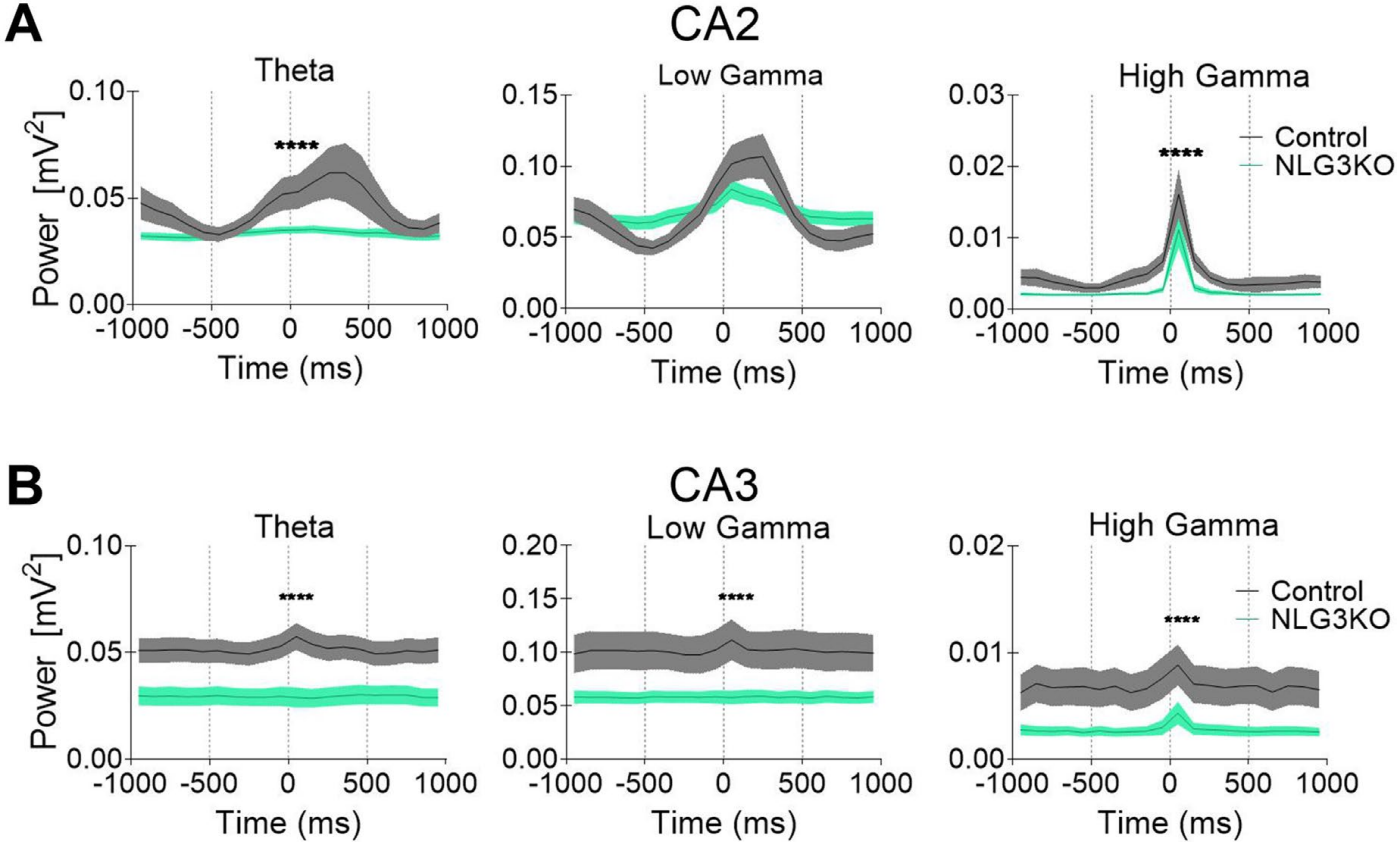
Reduced power in the theta and gamma frequency bands during spikes in NLG3 KO mice. **(A)** Summary plots showing relative power at theta (left), low gamma (middle), and high gamma (right) bands in the interval preceding (−1 s) and following (1 s) the occurrence of a spike in control (gray, *n* = 15535) and in NLG3 KO mice (green, *n* = 26092) in the CA2 region. **(B)** Same as A but for the CA3 region (control, *n* = 34804) and in NLG3 KO, *n* = 10875). *N* = spikes extracted from LFP, shaded areas: SEM.****p < 0.0001, Mann–Whitney test.

To dissect out possible alterations in neural oscillations, observed in several neurodevelopmental disorders (39, 40), raw LFP data were used to extract various bands occurring in the theta (5–14 Hz), low gamma (25–55 Hz), and high gamma (56–120 Hz) frequencies with Ensemble Empirical Mode Decomposition algorithm. While high-gamma oscillations are thought to contribute to memory encoding (41), low-gamma ones are believed to promote memory retrieval (42). To assess the average network activity, we measured power using the Welch Power Spectrum method and analyzed average relative power in individual LFP bands. In order to normalize for variations in raw LFP across animals and genotypes, we considered the relative power in each LFP band with respect to its corresponding raw LFP. This allowed us to compare normalized power across genotypes. We found a significant reduction in the average relative power of both low gamma and high gamma bands in the CA2 region of NLG3 KO mice as compared to controls (**Figures 3A, B**; theta: 0.045 ± 0.002 mV^2^ and 0.033 ± 0.001 mV^2^, *p* = 0.1; low gamma: 0.08 ± 0.003 mV^2^ and 0.06 ± 0.001 mV^2^, *p* < 0.0001; high gamma: 0.005 ± 0.0003 mV^2^ and 0.002 ± 0.0002 mV^2^, *p* < 0.0001 in controls and in NLG3 KO, respectively; Mann–Whitney test). Interestingly, also the CA3 region of NLG3 KO mice showed a reduced power in gamma as well as in theta bands (**Figures 3C, D**; theta: 0.057 ± 0.002 mV^2^ and 0.038 ± 0.002 mV^2^, *p* < 0.0001; low gamma: 0.11 ± 0.005 mV^2^ and 0.068 ± 0.003 mV^2^, *p* < 0.0001; high gamma: 0.008 ± 0.0006 mV^2^ and 0.003 ± 0.0002 mV^2^, *p* < 0.0001 in controls and in NLG3 KO, respectively; Mann–Whitney test). To assess whether the reduction in power relied on alterations in band occurrence frequency, the latter was quantified for each LFP bands in both CA2 and CA3 hippocampal regions. We found a significant increase in band occurrence frequency of high gamma band in both CA2 and CA3 regions of NLG3 KO mice as compared to controls. No significant alterations in band occurrence frequency of theta and low gamma rhythms of either regions were observed (**Figures S3A and B**; theta: 2.5 ± 0.16 Hz and 0.3 ± 0.12 Hz, *p* = 0.17; low gamma: 11.01 ± 0.17 Hz and 10.5 ± 0.2 Hz, *p* = 0.06; high gamma: 22.0 ± 1.1 Hz and 25.4 ± 0.7 Hz, *p* = 0.01 in controls and in NLG3 KO mice, respectively; Mann–Whitney test). These data suggest that the occurrence frequency of theta and low gamma are not responsible for reduction in power in theta and low gamma bands observed in NLG3 KO mice. To understand how theta and gamma power vary in CA3 and CA2 regions before and after a spike, we extracted spikes from raw LFP using the WaveClus, an unsupervised spike-sorting algorithm. Spike times were extracted, and LFP power, in and between theta and gamma bands, were analyzed 1 s before and after a spike (**Figure 4**). We found a significant reduction in power before spikes in theta and fast gamma bands in the CA2 region of NLG3 KO mice as compared to controls (**Figure 4A**; theta: 0.04 ± 0.002 mV^2^ and 0.03 ± 0.0002 mV^2^, *p* < 0.0001; low gamma: 0.1 ± 0.01 mV^2^ and 0.07 ± 0.005 mV^2^, *p* = 0.019; high gamma: 0.004 ± 0.0006 mV^2^ and 0.002 ± 0.0004 mV^2^, *p* < 0.0001 in controls and in NLG3 KO, respectively; Mann–Whitney test). In agreement with the reduction in power in both theta and gamma bands of NLG3 KO, we also found a significant decrease in power before spikes in all bands of CA3 region in NLG3 KO mice (**Figure 4B**; theta: 0.05 ± 0.0004 mV^2^ and 0.02 ± 0.0001 mV^2^, *p* < 0.0001; low gamma: 0.1 ± 0.0006 mV^2^ and 0.05 ± 0.0001 mV^2^, *p* < 0.0001; high gamma: 0.006 ± 0.0001 mV^2^ and 0.002 ± 8.6e^−5^ mV^2^, *p* < 0.0001 in controls and in NLG3 KO respectively; Mann–Whitney test) suggesting altered dynamics in both CA2 and CA3 network activities.

### Increased Bursting Activity in *In Vivo* Recordings From CA2 Principal Cells of NLG3 KO Mice

**Figure 5:**
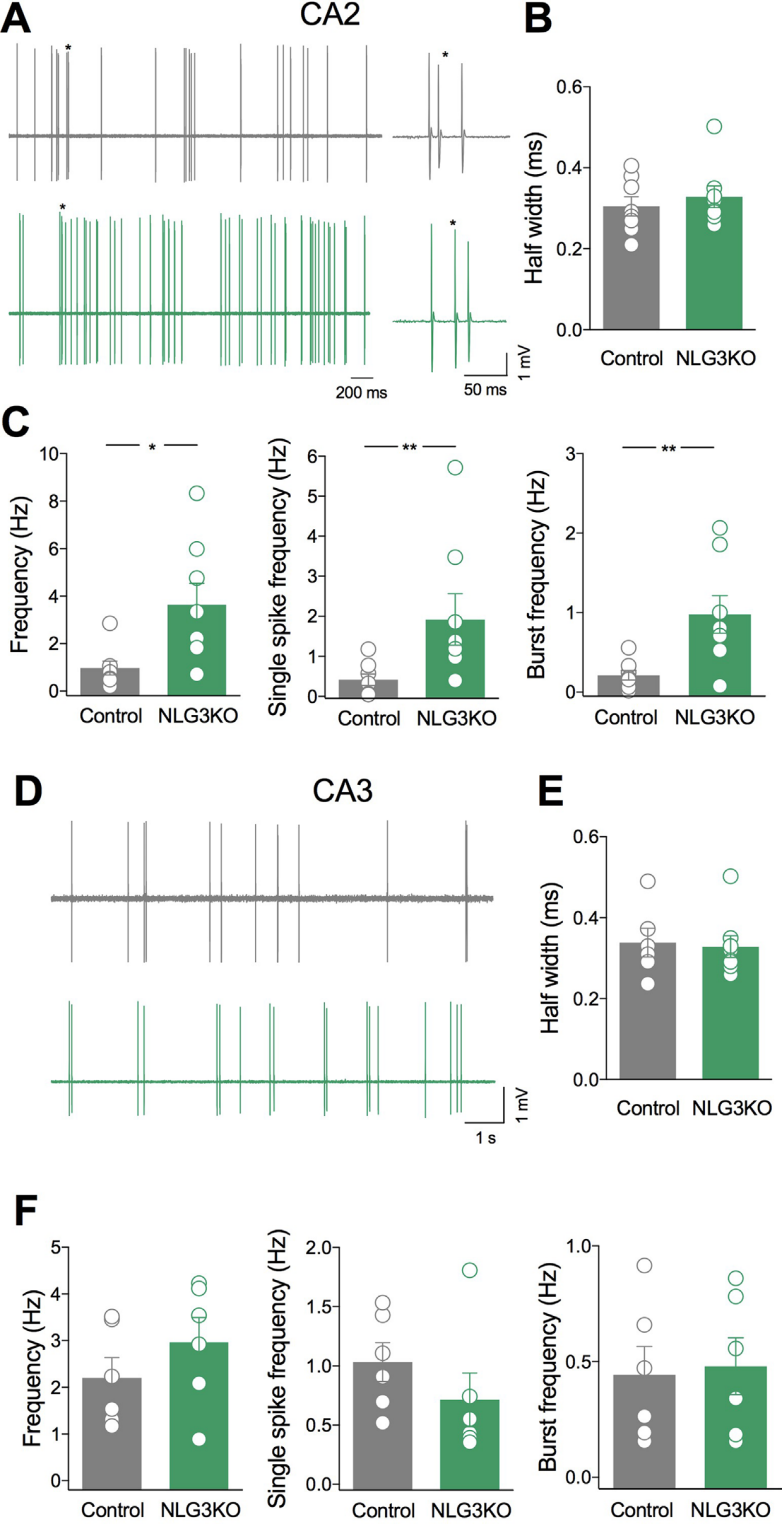
Selective enhancement of CA2 principal cells firing in NLG3 KO mice. **(A)** Sample traces of bursting neurons from a control (gray) and a NLG3 KO mouse (green) in the CA2 region. On the right individual bursts showed on an expanded time window (asterisks) **(B–C)** Scatter plots showing half spike width **(B)**, total, single spike frequency and burst frequency **(C)** of CA2 principal cells in control (gray, *n* = 8) and in NLG3 KO mice (green, *n* = 8). *p < 0.02; **p < 0.001, Mann–Whitney test). **(D)** Sample traces of bursting neurons from a control (gray) and a NLG3 KO mouse (green) in the CA3 region. **(E–F)** Scatter plots showing half spike width **(E)**, total single spike frequency and burst frequency **(F)** of CA3 principal cells in control (gray, *n* = 6) and in NLG3 KO mice (green, *n* = 6). Open circles represent values from single animals, and bars are mean ± SEM.

To understand whether alterations of network activity observed in the CA2 and CA3 hippocampal regions of NLG3 KO mice were associated to changes in their output, single neurons firing was recorded in *in vivo* anesthetized animals. Based on spontaneous firing frequency, firing mode and spike waveform, two populations of neurons could be clearly recognized in juxtacellular recordings from single CA2 neurons, corresponding to putative interneurons and pyramidal cells, respectively (43). While interneurons preferentially fire narrow action potentials at high rate, principal cells fire single action potentials intermixed with short duration bursts. Since interneurons are highly heterogeneous, we restricted the comparison between NLG3 KO and control mice to bursting neurons. The spontaneous firing [monitored for 5–10 min (**Figure 5A**)] and spike properties (i.e., half width, firing rate, bursting behavior) were evaluated in both controls and NLG3 KO mice. Similar half width were observed in CA2 neurons recorded from NLG3 KO and control mice suggesting that we targeted the same population of neurons (**Figure 5B**; NLG3 KO: 0.33 ± 0.3 ms and 0.3 ± 0.02 ms in controls and NLG3 KO mice, respectively; *p* = 0.59, Mann–Whitney test). A significant increase in the frequencies of total spikes (within and out of burst, **Figure 5C**, left; 0.97 ± 0.3 Hz and 3.6 ± 0.9 Hz in controls and NLG3 KO mice, respectively; *p* = 0.01, Mann–Whitney test), single spikes (**Figure 5C**, middle; 0.4 ± 0.1 Hz and 1.9 ± 0.6 Hz, in controls and NLG3 KO mice, respectively; *p* = 0.007, Mann–Whitney test), and bursts (**Figure 5C**, right; 0.2 ± 0.06 Hz and 0.98 ± 0.2 Hz in controls and NLG3 KO mice, respectively; *p* = 0.007, Mann–Whitney test) were observed in NLG3 KO mice as compared to controls. No differences in spike frequency within single bursts were observed (controls: 92.4 ± 8.8 Hz; NLG3 KO: 74.3 ± 12 Hz; *p* = 0.38, Mann–Whitney test). Similar half widths were observed in CA3 neurons recorded from NLG3 KO and control mice suggesting that we targeted the same population of neurons (**Figures 5D, E**; controls: 0.33 ± 0.3 ms; NLG3 KO: 0.3 ± 0.02 ms; *p* = 0.59, Mann–Whitney test). Interestingly, no changes in the firing frequency of CA3 bursting neurons were observed (**Figures 5D, F**) indicating that the lack of NLG3 causes a selective enhancement of neuronal excitability in the CA2 hippocampal area, known to process information relevant for social cognition.

### An Excitatory/Inhibitory Imbalance May Account for Altered Network Activity in the CA2 Region of the Hippocampus of NLG3 KO Mice

To understand whether the enhanced firing rate could be related to an excitatory/inhibitory imbalance within the CA2 hippocampal region, spontaneous excitatory and inhibitory postsynaptic currents mediated by AMPA and GABA_A_ receptors, respectively, were recorded in *ex vivo* slices obtained from controls and NLG3 KO mice.

**Figure 6:**
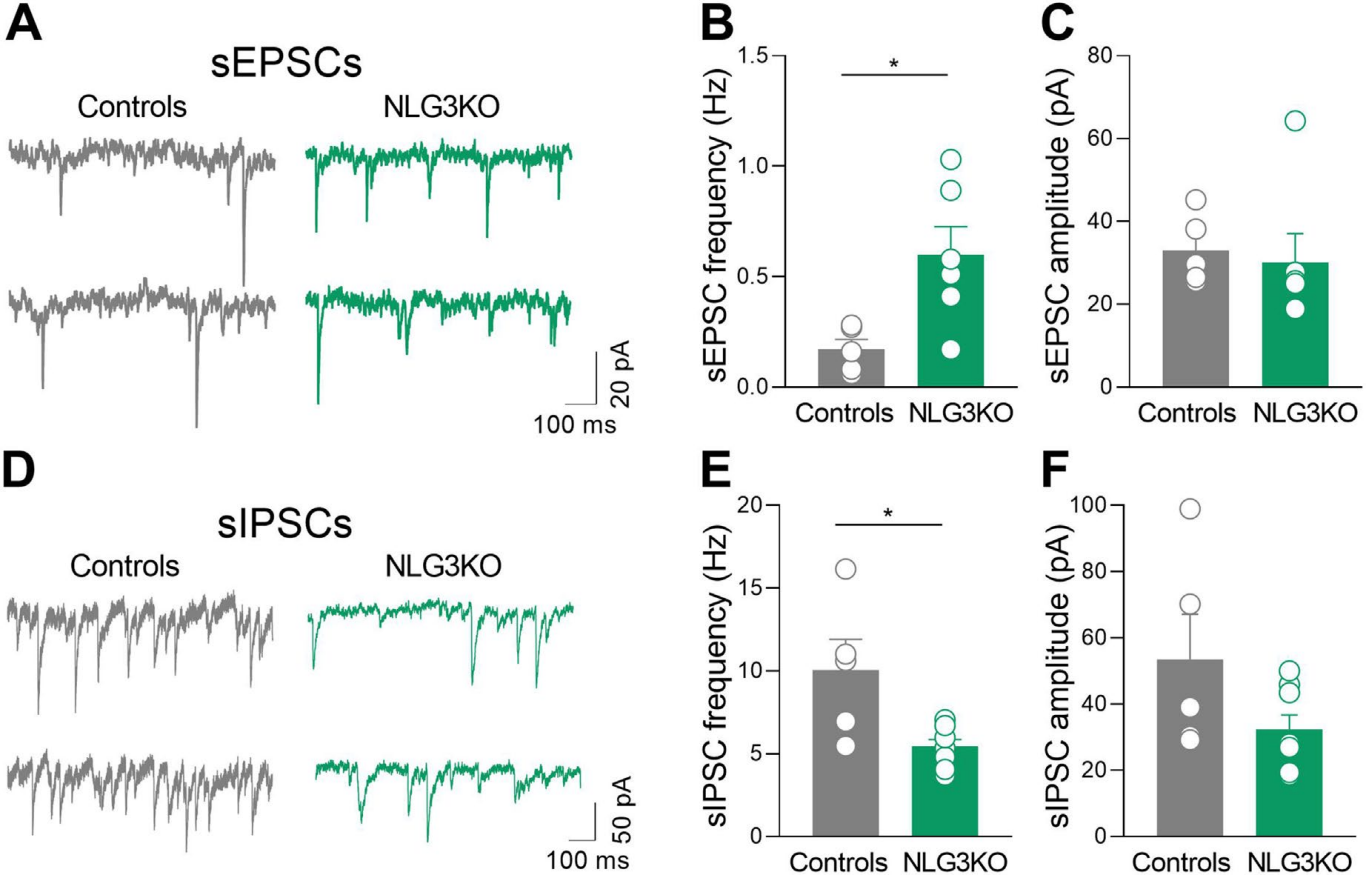
Enhanced excitatory and reduced inhibitory postsynaptic transmission in the CA2 region of NLG3 KO mice. **(A)** Sample traces showing sEPSCs from CA2 pyramidal neurons in hippocampal slices obtained from a control (gray) and a NLG3 KO mouse (green). **(B, C)** Scatter plots showing the mean frequency **(B)** and amplitude **(C)** of sEPSC recorded from CA2 pyramidal neurons in control (gray, *n* = 6) and in NLG3 KO mice (green, *n* = 5). Bars represent SEM; open circles represent values from single cells. **(D)** Sample traces showing sIPSCs from CA2 pyramidal neurons in hippocampal slices obtained from a control (gray) and a NLG3 KO mouse (green). **(E, F)** Scatter plots showing the mean frequency **(E)** and amplitude **(F)** of sIPSC recorded from CA2 pyramidal neurons in control (gray, *n* = 6) and in NLG3 KO mice (green, *n* = 5). Bars represent SEM. Open circles represent values from single cells. *p < 0.05, Mann–Whitney test.

A significant increase in frequency (**Figures 6A, B**; controls: 0.17 ± 0.05 Hz and NLG3 KO: 0.6 ± 0.13 Hz; *p* = 0.017, Mann–Whitney test) but not in amplitude (**Figures 6A, C**; controls: 33 ± 3.8 pA, NLG3 KO 30.1 ± 7 pA; *p* = 0.25, Mann–Whitney test) of sEPSCs, recorded from CA2 principal cells in the presence of picrotoxin, was found in NLG3 KO as compared to controls. This effect was associated to a significant decrease in frequency (but not in amplitude) of sIPSCs recorded in the presence of CNQX and DL-APV (**Figures 6D, E**; controls: 10 ± 1.8 Hz, NLG3 KO: 5.4 ± 0.4 Hz; *p* = 0.03, Mann–Whitney test) but not in amplitude (**Figures 6D–F**; controls: 53.6 ± 13.6 pA, NLG3 KO: 32.4 ± 4.3 pA; *p* = 0.13, Mann–Whitney test).

These results strongly suggest that an excitatory/inhibitory imbalance within the CA2 hippocampal region may be instrumental for the *in vivo* dynamic changes observed in NLG3 KO mice.

### Impairment of Perisomatic Inhibition Mediated by GABAergic Interneurons Expressing CCK in NLG3 KO Mice

To determine whether alterations in network oscillations observed in *in vivo* and in *ex vivo* recordings from NLG3 KO mice are due to impairments of perisomatic inhibition, which efficiently suppresses repetitive sodium-dependent action potentials (44) and entrains network oscillations (45), inhibitory synaptic currents evoked in CA2 pyramidal cells by stimulation of GABAergic inputs were analyzed. Stimulation of GABAergic inputs, with an intensity equal to 60% of that necessary to evoke a maximal response, induced monosynaptic IPSCs. IPSCs showed similar rise (controls: 4.89 ± 2 ms, NLG3 KO: 3.3 ± 1.6 ms; *p* = 0.6, Mann–Whitney test) and decay times (controls: 31.1 ± 2.4 ms, NLG3 KO: 26.2 ± 3.7 ms; *p* = 0.2, Mann–Whitney test) in both genotypes.

**Figure 7:**
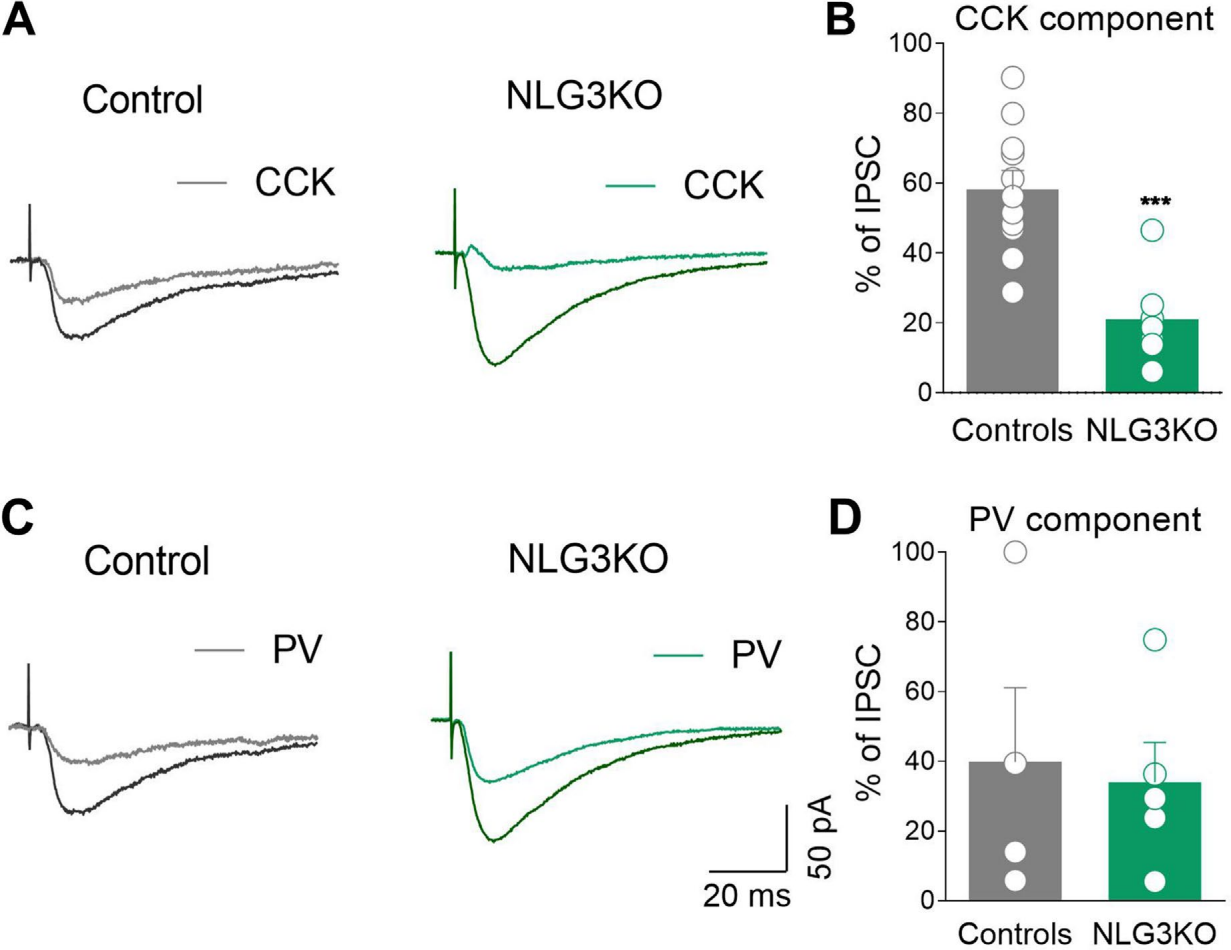
Reduced CCK-mediated inhibition in CA2 region of NLG3 KO mice. **(A)** Sample traces of IPSCs evoked in CA2 pyramidal neurons by stimulation of GABAergic inputs in the pyramidal layer in control and in NLG3 KO mice before (black and dark green, respectively) and after (gray and light green, respectively) application of ω-Ctx-GVIA 1 µM. **(B)** Summary plot showing the percentage amplitude of CCK-mediated responses (ω-Ctx-sensitive currents) in control (gray, *n* = 11) and in NLG3 KO mice (green, *n* = 7). **(C)** Sample traces of IPSCs evoked in CA2 pyramidal neurons by stimulation of GABAergic inputs in the pyramidal layer in control and in NLG3 KO mouse before (black and dark green, respectively) and after (gray and light green, respectively) application ω-Agtx-IVA 300 nM. **(D)** Summary plot showing the percentage amplitude of PV-mediated responses (ω-Agtx-IVA-sensitive currents) in control (gray, *n* = 4) and in NLG3 KO mice (green, *n* = 5). Open circles represent values from single cells, and bars are mean ± SEM. ***p = 0.0003; Mann–Whitney test.

GABAergic terminals present in the pyramidal layer belong mainly to CCK+ and PV+ GABAergic interneurons (46). Interestingly, GABA release from these two interneuron subtypes is triggered by calcium entry *via* different voltage-gated calcium channels, N types, and P/Q, respectively, sensitive to different toxins. While the release of GABA from CCK+ interneurons is selectively blocked by ω-Ctx-GVIA, that from PV+ ones is selectively blocked by ω-Agtx-IVA (25, 26, 47). Taking advantage of these two toxins, we found that while the CCK component of the evoked IPSC (sensitive to ω-Ctx-GVIA, 1 μM) was strongly reduced in NLG3 KO mice as compared to controls (**Figure 7A, B**, controls: 58.2 ± 5.5%, NLG3 KO: 21 ± 4.8%; *p* = 0.0003, Mann–Whitney test), the PV component (sensitive to ω-Agtx-IVA, 300 nM) was unaltered in both genotypes (**Figures 7C, D**; controls: 39.8 ± 21%, NLG3 KO: 34 ± 11%; *p* = 0.9, Mann–Whitney test).

To study whether the lack of NLG3 alters inhibitory short-term plasticity, GABAergic interneurons were stimulated with brief train of five stimuli at 20 Hz. As previously shown for somatic targeting GABAergic synapses (48) this stimulation paradigm led to a similar short-term depression in both NLG3 KO and control mice (**Figure S4**, ratio last/first response, controls: 0.48 ± 0.08, NLG3 KO: 0.28 ± 0.04;* p* = 0.1, Mann–Whitney test), indicating that the lack of NLG3 does not affect GABAergic short-term plasticity. As expected, application of ω-Ctx-GVIA strongly reduced the amplitude of IPSCs, without altering in both genotypes the degree of depression of the remaining components (**Figure S4**, ratio last/first response; controls: 0.58 ± 0.2, NLG3 KO: 0.3 ± 0.1; *p* = 0.65, Mann–Whitney test). These data clearly indicate that deficits in basal GABAergic signaling *via* CCK+ interneurons contribute to enhance cell excitability and to alter network dynamics in the CA2 region of the hippocampus.

### Loss of NLG3 Does Not Significantly Affect the CCK- and PV-Mediated Innervation of the CA2 Hippocampal Region

**Figure 8:**
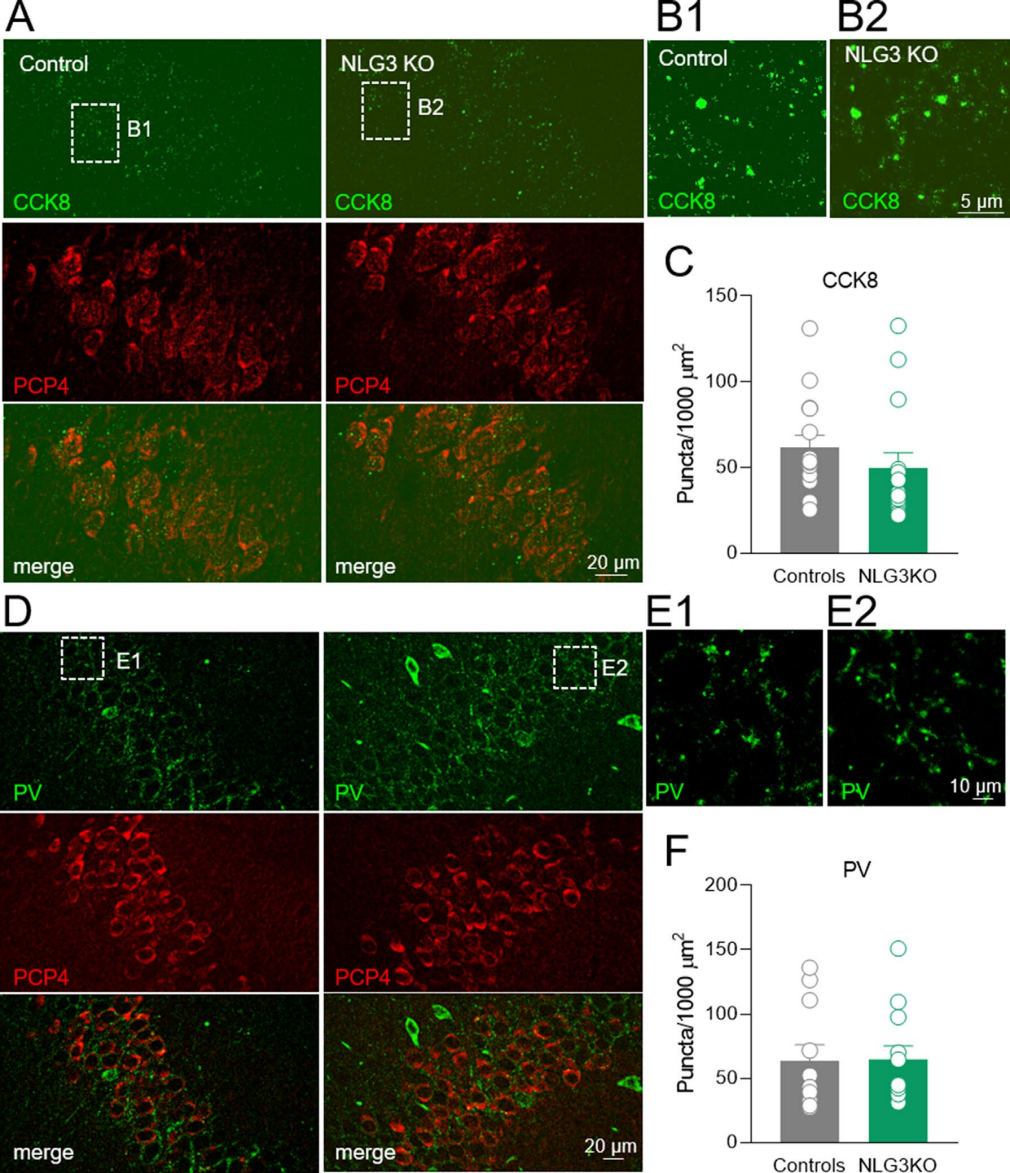
Loss of NLG3 does not significantly affect GABAergic innervation from CCK and PV positive interneurons in the CA2 hippocampal region. **(A)** Double immunostaining labeling the presynaptic CCK terminals (CCK8) and CA2 principal neuron (PCP4) in control and NLG3 KO mice. **(B1–B2)** insets of **(A)** showing higher magnification of CCK positive terminals in control and NLG3 KO mice. **(C)** Summary plot showing the number of CCK positive puncta in 1,000 µm^2^ (*n* = 15 from three animals per genotype). Open circles represent values from CA2 hippocampal single section analyzed, and bars are mean ± SEM. *p* = 0.07; Mann–Whitney test. **(D–F)** as for **(A–C)** but for PV positive interneurons (*n* = 11 from three controls and *n* = 12 from three NLG3 KO mice).

To assess whether a reduction in CCK innervation could play a role in the decreased CCK-mediated current observed in NLG3 KO mice, we performed immunohistochemical analysis of CCK terminals in the CA2 pyramidal layer using a CCK marker (49). Although a tendency toward a reduction of CCK positive puncta in NLG3 KO mice was observed as compared to controls (**Figures 8A–C**; controls: 61.6 ± 7.3, NLG3KO: 49.8 ± 8.8; *p* = 0.07, Mann–Whitney test), this did not reach statistical significance. This result suggests that a reduced probability of GABA release from CCK terminals is mainly responsible for the reduced amplitude of CCK-mediated current in mice lacking NLG3. In agreement with electrophysiological data, no differences in the number of PV positive puncta measured in the principal layer of CA2 region were observed (**Figures 8D–F**; controls: 63.5 ± 12, NLG3KO: 64.7 ± 11; *p* = 0.6, Mann–Whitney test).

## Discussion

Here we provide evidence that the NLG3 KO mouse, an animal model of non-syndromic form of autism, exhibits clear alterations in network dynamics in the CA2 region of the hippocampus, probably at the origin of social memory defects reminiscent of those found in autistic patients. In agreement with previous studies (17, 38), NLG3-deficient mice did not show any preference for the novel mouse in the social novelty test, suggesting an impairment in the capacity to remember a conspecific, an effect associated to an enhanced exploratory behavior in the open field. However, in contrast with Radyushkin et al., (17), NLG3 KO mice exhibited clear sociability deficits as assessed by the reduced time in exploring the animal as compared to their control littermates and by the lack of preference for the animal *versus* the object. This discrepancy may be due to the fact that in contrast with Radyushkin et al., (17), who have used as controls age-matched C57Bl/6 male, we have employed littermates reared, as NLG3 KO, in the same cage, a condition known to critically affect, through peers interactions, the acquisition of social behavior (50).

To identify the circuitry responsible for social deficits in ASDs represents a key step for understanding the pathophysiology of these disorders. Several brain areas encode social behavior, including the medial prefrontal cortex (51), the basolateral amygdala (52), and the CA2 region of the hippocampus (20, 53). Our *in vivo* experiments from NLG3 KO mice clearly demonstrate selective changes in the amplitude of slow waves preceding or following the occurrence of spikes (spike-triggered average or STA). These modifications reflect alterations in electrophysiological signals produced by large neuronal ensembles in that specific region of that particular genotype and therefore cannot be attributed to the anesthetic used. LFPs represent summed synaptic activity located in small volume around the recording site, related to the strength of functional connectivity between neurons (54). Consistent with STA data, the observed modifications in theta and gamma power probably reflect aberrant functional connectivity between the hippocampus and associated networks (55–57). However, while STA defects were confined to the CA2 region of the hippocampus, changes in theta and gamma power occurred in both CA2 and CA3 hippocampal areas. This is in accord with the recent observation that both CA2 and CA3 hippocampal regions contribute to generate gamma oscillations (58). Changes in theta and gamma power have been often found in the EEG/MEG of autistic children during their resting state (59–63) or during sustained attention (64). Interestingly, as in the present experiments, a strong reduction in gamma power associated with a dysfunction of GABAergic signaling was detected in the CA3 region of the hippocampus of mice lacking the adhesion molecule NLG4, exhibiting behavioral deficits reminiscent of those found in ASDs (65). However, unlike the present experiments, in NLG4 knock-out mice the oscillatory activity pharmacologically induced by kainate *in vitro* was highly dependent on perisomatic inhibition generated by PV+ interneurons.

As in the present experiments, abnormal oscillatory activity may arise from elevation in the cellular balance of excitation and inhibition, *via* an increased excitatory and a decreased inhibitory activity (66). The E/I balance represents a critical condition for the correct functioning of neuronal networks, and it is essential for nearly all brain functions, including representation of sensory information and cognitive processes (67). In the present study, the E/I imbalance accounts for the selective enhancement of cell excitability recorded in *in vivo* experiments from the CA2 hippocampal region. It is commonly accepted that network oscillations generated by the interplay between excitation and inhibition involve the activity of PV+ interneurons mediating the feedforward and feedback inhibition (68). Unexpectedly, a more detailed analysis into perisomatic inhibition, using toxins known to affect presynaptic calcium rise *via* VDCC, has unveiled a clear reduction in the probability of GABA release from CCK+ interneurons in the CA2 hippocampal region. Interestingly, in contrast with the present data, a detailed analysis of unitary inhibitory postsynaptic currents between CCK+ interneurons and principal cells in the CA1 region of the hippocampus revealed an increased probability of GABA release probably consequent to impairment of a tonic endocannabinoid signaling (23), suggesting that, in mice lacking NLG3, changes in GABAergic signaling are cell and circuit specific. Although PV+ and CCK+ interneurons target the same domains of pyramidal cells, the functional role of the latter in network synchronization is still unclear. By comparing, in anesthetized rats, spike time during oscillations, Klausberger et al. (69) suggest that these interneuron subtypes contribute differently to network activity. While PV expressing interneurons provide more stereotyped oscillatory entrainment of the entire network, CCK+ interneurons might shape the activity of subgroups of principal cells forming neuronal assemblies.

In conclusion, our data from NLG3 mice strongly suggest that alterations in CA2 network dynamics may account for the behavioral deficits, similar to those observed in autistic children. It should be stressed however that, while social memory is likely to be processed by the CA2 region of the hippocampus, sociability might involve other brain structures including the basolateral complex of the amygdala, which projects to the ventral hippocampus (52).

Whatever the origin of behavioral deficits reported here, our results further highlight the crucial role of an altered GABA_A_-mediated neurotransmission in neurodevelopmental disorders (67, 70). Thus, interfering with GABAergic signaling may be considered as a key strategy for the treatment of some forms of ASDs that involve genetic modification/deletions of synaptic proteins.

## Data Availability

All datasets generated for this study are included in the manuscript and the supplementary files.

## Ethics Statement

This study was carried out in accordance with principles of Basel declaration and recommendations of Italian Animal Welfare legislation (D.L. 26/2014) that implemented the European Committee Council Directive (2010/63 EEC). The protocol was approved by local veterinary authorities, the EBRI ethical committee and the Italian Ministry of Health (authorization number: 1084/PR15).

## Author contributions

MG and EC designed the research with the contribution of all authors. DP performed behavioral experiments. BM and DP performed slice electrophysiology. BM and AP performed analysis of *in vivo* data. MG performed *in vivo* electrophysiology and immunohistochemistry. PZ performed WB. All authors participated in the interpretation of data. EC and MG wrote the manuscript with comments and contributions from all authors.

## Funding

This work was supported by grants from Telethon (GGP 16083) to EC, from the Veronesi’s Foundation (Postdoctoral Fellowship to MG), from the European Union’s Horizon 2020 Framework Programme for Research and Innovation under the Specific Grant Agreement No. 785907 (Human Brain Project SGA2) to EC.

## Conflict of Interest Statement

The authors declare that the research was conducted in the absence of any commercial or financial relationships that could be construed as a potential conflict of interest.

## Supplementary Material

Figure S1 Lack of NLG3 protein in the hippocampus of NLG3 KO mice. Immunoblot analysis of NLG3 expression in the hippocampus of controls and NLG3 KO mice. Western blot detecting actin was used as loading control.

Figure S2 Enhanced locomotor activity in NLG3 KO mice. **(A)** Examples of track plots showing locomotor activity during the open field task in a control (left) and in a NLG3 KO mouse (right). **(B)** Scatter plots showing the total distance traveled by control (gray, n = 10) and NLG3 KO mice (green, n = 9). **(C)** Scatter plot showing the velocity in control (gray, n = 10) and in NLG3 KO mice (green, n = 9). **(D)** Scatter plots showing the time spent by control (gray, n = 10) and NLG3 KO mice (green, n = 9) in the inner and outer zones, in the open field test. Open circles represent values from single animals, and bars are mean ± SEM.

Figure S3 Enhanced high gamma occurrence in NLG3 KO mice. **(A)** Summary plots showing theta (left), low gamma (middle), and high gamma (right) occurrence frequency in the CA2 region of control (gray, n = 17) and NLG3 KO mice (green, n = 14). **(B)** Same as **(A)** but for the CA3 area (control: n = 15; NLG3 KO, n = 11). Bars are SEM. **p*< 0.05, Mann-Whitney test.

Figure S4 No changes in the short-term GABAergic plasticity in NLG3 KO mice. **(A)** Sample traces of five consecutive IPSCs evoked at 20 Hz in CA2 pyramidal cells by stimulation of GABAergic inputs in the pyramidal layer in control and in NLG3 KO mice before (grey and green, respectively) and after (black) application of ω-Ctx-GVIA 1 µM. **(B)** Scatter plots showing mean IPSC amplitudes (± SEM) evoked at 20 Hz in CA2 principal cells in control (n = 5) and in NLG3 KO mice (*n* = 4) before (grey and green, respectively) and after (black) application of ω-Ctx-GVIA 1 µM. Open circles are mean ± SEM.
