# Supplementary figures and images for: Possible Implication of the CA2 Hippocampal Circuit in Social Cognition Deficits Observed in the Neuroligin 3 Knock-Out Mouse, a Non-Syndromic Animal Model of Autism
Source: Front Psychiatry. 2019 Jul 19;10:513. doi: 10.3389/fpsyt.2019.00513 (PMC6659102; doi:10.3389/fpsyt.2019.00513)

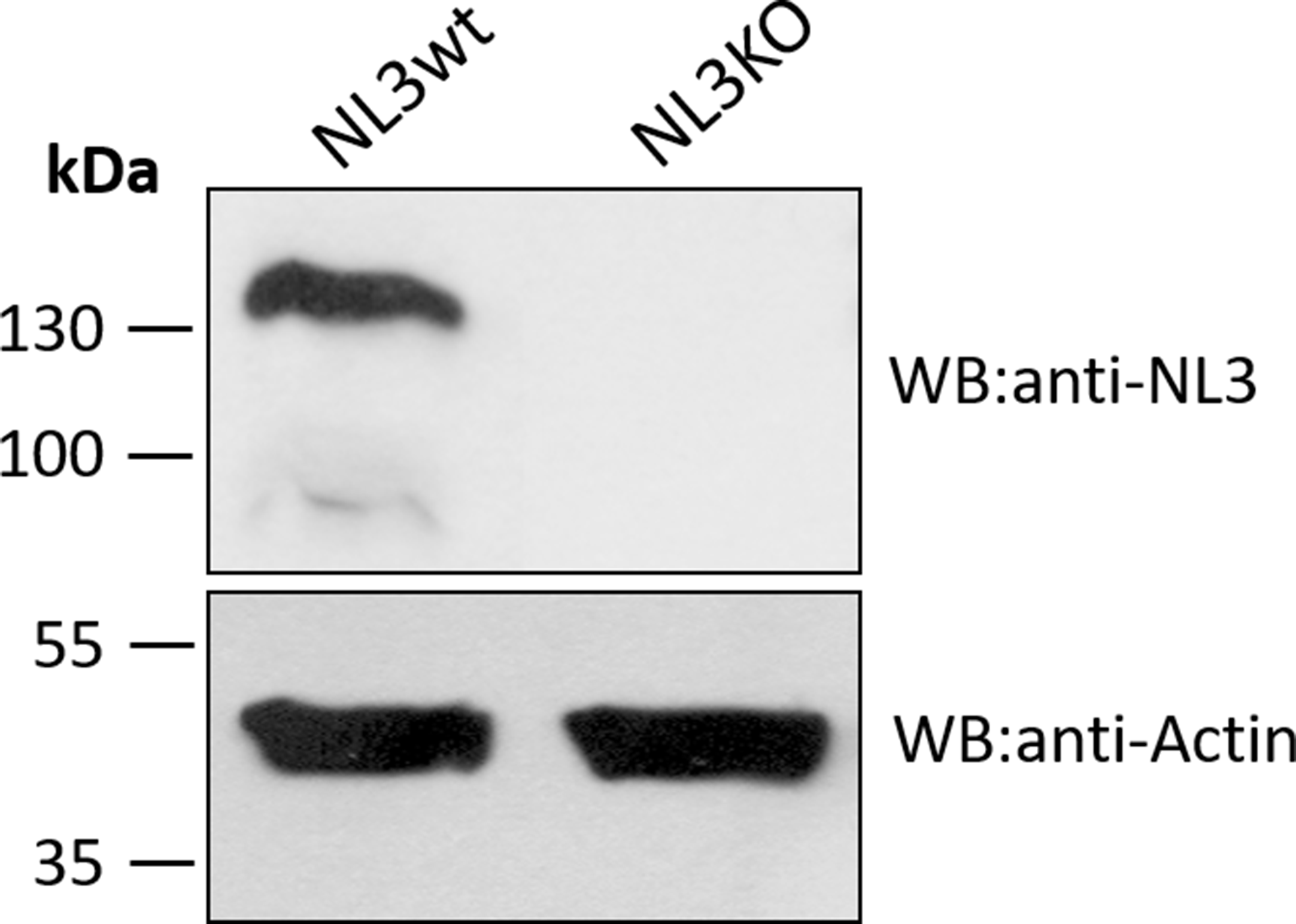

Supplement: Figure S1 — Lack of NLG3 protein in the hippocampus of NLG3 KO mice. Immunoblot analysis of NLG3 expression in the hippocampus of controls and NLG3 KO mice. Western blot detecting actin was used as loading control. [file Image_1.tif]

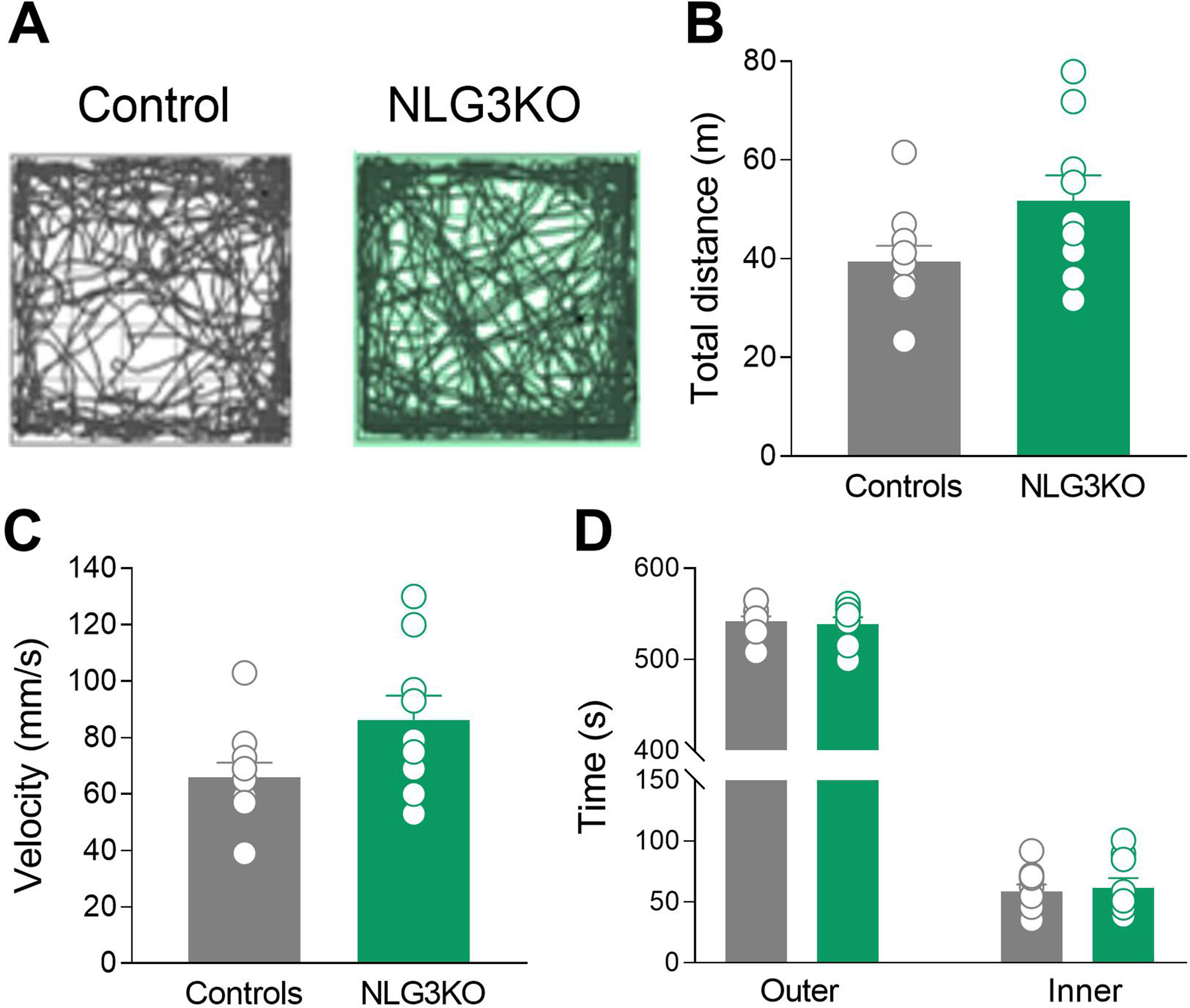

Supplement: Figure S2 — Enhanced locomotor activity in NLG3 KO mice. (A) Examples of track plots showing locomotor activity during the open field task in a control (left) and in a NLG3 KO mouse (right). (B) Scatter plots showing the total distance traveled by control (gray, n = 10) and NLG3 KO mice (green, n = 9). (C) Scatter plot showing the velocity in control (gray, n = 10) and in NLG3 KO mice (green, n = 9). (D) Scatter plots showing the time spent by control (gray, n = 10) and NLG3 KO mice (green, n = 9) in the inner and outer zones, in the open field test. Open circles represent values from single animals, and bars are mean ± SEM. [file Image_2.tif]

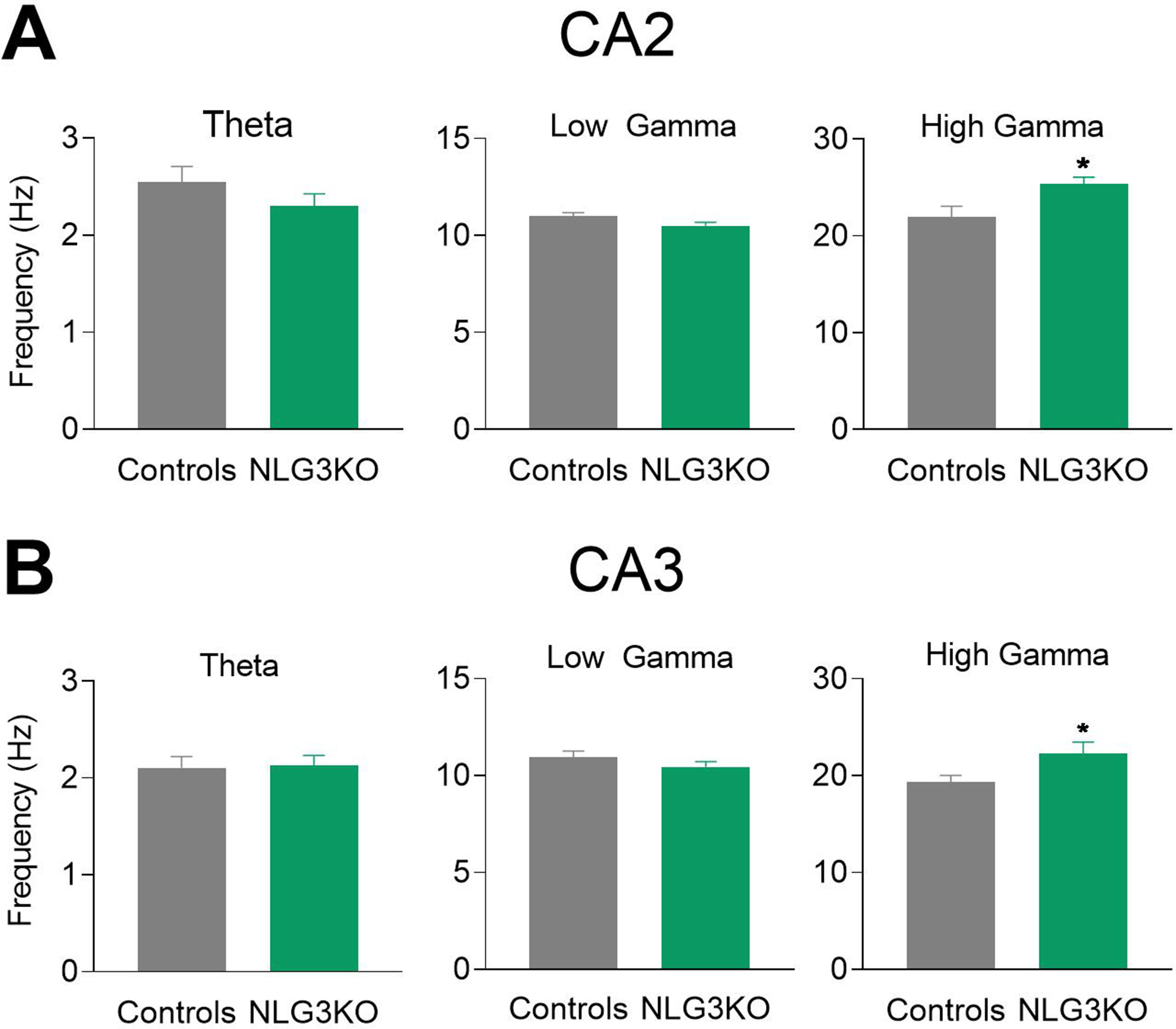

Supplement: Figure S3 — Enhanced high gamma occurrence in NLG3 KO mice. (A) Summary plots showing theta (left), low gamma (middle), and high gamma (right) occurrence frequency in the CA2 region of control (gray, n = 17) and NLG3 KO mice (green, n = 14). (B) Same as (A) but for the CA3 area (control: n = 15; NLG3 KO, n = 11). Bars are SEM. *p< 0.05, Mann-Whitney test. [file Image_3.tif]

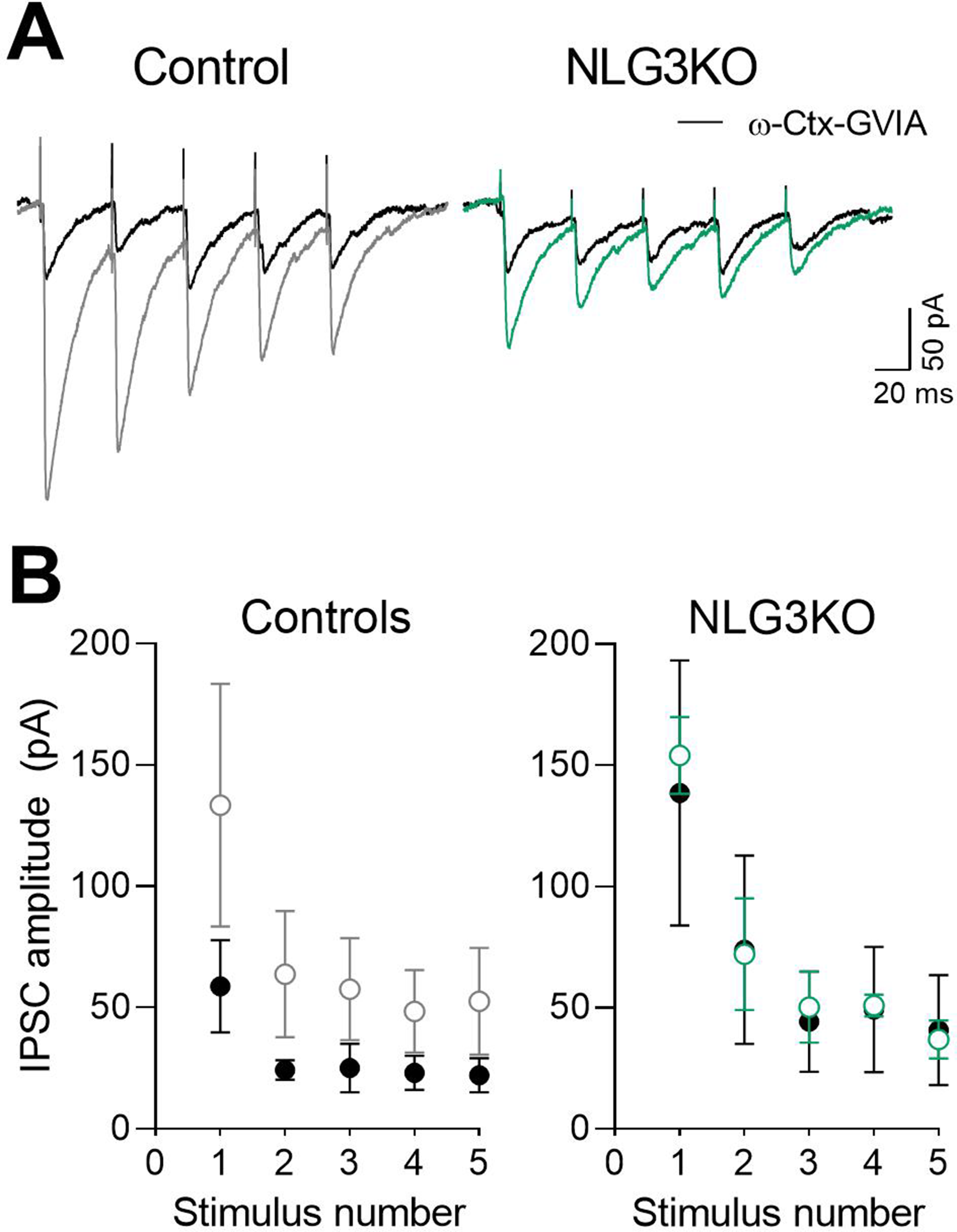

Supplement: Figure S4 — No changes in the short-term GABAergic plasticity in NLG3 KO mice. (A) Sample traces of five consecutive IPSCs evoked at 20 Hz in CA2 pyramidal cells by stimulation of GABAergic inputs in the pyramidal layer in control and in NLG3 KO mice before (grey and green, respectively) and after (black) application of ω-Ctx-GVIA 1 µM. (B) Scatter plots showing mean IPSC amplitudes (± SEM) evoked at 20 Hz in CA2 principal cells in control (n = 5) and in NLG3 KO mice (n = 4) before (grey and green, respectively) and after (black) application of ω-Ctx-GVIA 1 µM. Open circles are mean ± SEM. [file Image_4.tif]
